# Determination of 69 Pesticide Residues in 42 Batches of Platycodonis Radix and Dietary Risk Assessment Using Combined QuEChERS with GC-MS/MS and UHPLC-MS/MS

**DOI:** 10.3390/foods15111835

**Published:** 2026-05-22

**Authors:** Jing Ma, Xinyue Qiu, Suiqing Chen, Haibo Wang, Xiaoya Sun

**Affiliations:** 1 School of Pharmacy, Henan University of Chinese Medicine, Zhengzhou 450046, China; mj021017@163.com (J.M.); q_xinyue@163.com (X.Q.); suiqingchen0371@163.com (S.C.); 2 NMPA Key Laboratory for Quality Control of Traditional Chinese Medicine (Chinese Materia Medica and Prepared Slices), Zhengzhou 450018, China; 3 Collaborative Innovation Center of Research and Development on the Whole Industry Chain of Yu-Yao, Henan University of Chinese Medicine, Zhengzhou 450046, China; 4 Collaborative Innovation Center for Respiratory Disease Diagnosis and Treatment & Chinese Medicine Development of Henan Province, Henan University of Chinese Medicine, Zhengzhou 450046, China; 5 Henan Institute for Drug and Medical Device Inspection, Zhengzhou 450018, China; 6 Henan Province Engineering Research Center for Quality Control of Traditional Chinese Medicine, Zhengzhou 450018, China

**Keywords:** pesticide residues, Platycodonis Radix, QuEChERS, GC-MS/MS, UHPLC-MS/MS, risk assessment

## Abstract

This study aimed to establish a rapid analytical method for the determination of 69 pesticide residues in Platycodonis Radix using GC-MS/MS and UHPLC-MS/MS, as well as carry out a dietary risk assessment on 42 batches of Platycodonis Radix samples collected from different geographical origins. Samples were prepared using the QuEChERS method, followed by high-speed centrifugation and membrane filtration, and the target pesticides were analyzed in selected reaction monitoring (SRM) mode via GC-MS/MS and in multiple reaction monitoring (MRM) mode via UHPLC-MS/MS. Among the 42 tested batches of Platycodonis Radix samples, 3 out of 27 pesticide compounds were detected via GC-MS/MS screening, while only 1 pesticide compound was positive from the 42 compounds determined via UHPLC-MS/MS, and the risk assessment results demonstrated that both chronic and acute dietary exposure risks of all detected pesticides were considerably lower than 1.

## 1. Introduction

*Platycodon grandiflorus* (Jacq.) A. DC. (PG) is predominantly distributed across East Asia, including China, Korea, and Japan, and has also been introduced into cultivation in certain regions of Europe and North America [1]. Originating in China, wild populations of PG are widely distributed in Guangdong, Guizhou, Yunnan, Sichuan, and other provinces. Platycodonis Radix is the dried root of PG [2]. This broad geographical distribution has led to the classification of two primary varieties: Northern and Southern Platycodonis Radix [3]. At present, the core producing areas are concentrated in Chifeng (Inner Mongolia), Bozhou (Anhui), Zibo (Shandong), Shangluo (Shaanxi), and Song County in Luoyang (Henan) [4]. As a classical medicinal and edible herb, Platycodonis Radix holds significant value in both clinical therapy and the production of health-promoting foods. Modern pharmacological studies have not only validated its traditional antitussive and expectorant effects [5] but also elucidated its multifarious pharmacological activities, including anti-inflammatory [6], antioxidant [7], immunomodulatory [8], hepatoprotective [9], and antitumor properties [10]. In clinical practice, it is frequently employed in compound formulations for the treatment of respiratory diseases such as chronic bronchitis, pharyngitis, and pneumonia. Moreover, it exhibits certain therapeutic potential in the management of digestive disorders, cardiovascular diseases, and as an adjuvant in cancer therapy [11]. Furthermore, owing to its distinctive flavor and nutritional profile, Platycodonis Radix has been developed into a variety of commercial products in the food industry, including health-promoting teas, functional candies, fermented pickles, and functional beverages [12]. Nevertheless, with escalating market demand and the depletion of wild resources, artificial cultivation has become the primary means of supplying this herb for commercial purposes.

Platycodonis Radix exhibits a relatively prolonged growth cycle and thrives in moist soil conditions, yet remains susceptible to various pests and diseases during cultivation. Predominant diseases include root rot caused by Fusarium solani, leaf spot induced by Alternaria spp., and powdery mildew resulting from Erysiphe cichoracearum. Common insect pests encompass aphids, cutworms, and wireworms, among others [13]. To effectively manage these biotic stressors and ensure yield stability, growers frequently employ chemical pesticides such as organophosphates, pyrethroids, and triazoles. Although chemical interventions can rapidly suppress pest and disease outbreaks, excessive or improper application often leads to elevated pesticide residues in the plant material. This not only compromises the quality of the medicinal herb but also poses potential health risks to consumers through dietary exposure [14]. Consequently, pesticide residue has emerged as a critical factor constraining its safe utilization and the sustainable development of the Platycodonis Radix industry.

In the field of pesticide residue analysis, major international pharmacopeias such as the European Pharmacopoeia (EP 11.0) and the United States Pharmacopoeia (USP 46) have systematically established maximum residue limit standards (MRLs) for over 120 pesticides, including organophosphates, organochlorines, pyrethroids, and neonicotinoids [15]. Globally recognized MRLs specifically applicable to Platycodonis Radix are still lacking. The 2025 edition of the Pharmacopoeia of the People’s Republic of China sets general MRLs for 47 banned pesticides, including relevant metabolites and isomers, which are applicable to common herbal medicines. However, specific residue limits for routine quality supervision of Platycodonis Radix remain limited, underscoring the need for reliable multi-residue analytical methods to support comprehensive quality and safety evaluation.

The advancement of modern instrumental analysis technologies has significantly propelled the detection of pesticide residues in traditional Chinese medicines toward higher sensitivity, selectivity, and throughput. In terms of sample preparation, the QuEChERS method has emerged as the mainstream choice for multi-pesticide residue extraction and cleanup in complex matrices, owing to its simplicity, rapidity, and cost-effectiveness, making it particularly suitable for high-lipid and multi-interference herbal matrices [16]. At the detection level, GC-MS/MS and UHPLC-MS/MS have become core techniques for the quantitative analysis of pesticide residues in medicinal plants. Recent years have witnessed continuous improvements in their detection sensitivity and multi-component simultaneous analysis capabilities. For instance, Hu et al. employed UHPLC-MS/MS to successfully achieve the simultaneous separation and quantification of 30 pesticides in Astragalus [17]. In a study on Coix Seed, Huang et al. established a non-targeted screening method based on UHPLC-MS/MS, which, through matching with a database containing 1608 compounds, identified 39 potential pesticide residues, further validating the applicability of this technology for multi-residue screening in rhizome-based medicinal materials [18]. GC-MS/MS demonstrates distinct advantages in detecting non-polar and volatile pesticides and is often used complementarily with UHPLC-MS/MS. In research on pesticide residues in Ginseng, Xu et al. utilized a combined GC-MS/MS and UHPLC-MS/MS approach to systematically elucidate the migration and transformation patterns of 30 pesticides during processing, demonstrating that the combined use of both techniques enables comprehensive coverage of pesticide residue analysis in traditional Chinese medicines [19]. These analytical systems, refined through years of research and validation, have established standardized protocols for rhizome-based medicinal materials such as Astragalus, Ginseng, and Coix Seed, exhibiting good accuracy, reproducibility, and high technical maturity.

Previous studies on pesticide residue analysis in Platycodonis Radix have faced certain limitations. Conventional sample preparation techniques, such as gel permeation chromatography (GPC) and solid-phase extraction (SPE) [20,21], have been used for pesticide residue analysis in complex matrices. However, these procedures may require additional cleanup steps, longer processing time, or greater solvent and material consumption, which can limit their convenience for high-throughput multi-residue analysis. Many previous studies have relied on a single analytical platform, such as GC-MS/MS, GC-IT-MS, or GC-FPD, and often focused on a limited subset of pesticide classes, which may restrict the coverage of pesticides with diverse physicochemical properties [22]. These challenges can be further influenced by the complex chemical composition of Platycodonis Radix, which contains polysaccharides, saponins, inulin, and various secondary metabolites, potentially causing matrix effects during extraction and detection, and thereby affecting the performance of conventional sample preparation and single-platform analyses for multi-class pesticide residue determination.

Based on the aforementioned technological context and practical regulatory needs, this study systematically selected 69 prohibited pesticides as target analytes, covering multiple categories such as organochlorines and organophosphates, with reference to literature reports and pharmacopeial standards. A validated analytical workflow for multi-pesticide residues in Platycodonis Radix was established using a conventional QuEChERS sample preparation procedure. Previous studies have reported [23] the applicability of QuEChERS-based procedures for multi-residue pesticide analysis in various root and rhizome medicinal herbs; therefore, this procedure was selected in this study based on its reported applicability and operational convenience for complex plant matrices. This pretreatment method was further combined with GC-MS/MS and UHPLC-MS/MS. Notably, the two instrumental platforms were applied in a complementary manner, with distinct and non-overlapping target compounds, allowing full utilization of their respective applicability: GC-MS/MS for volatile and semi-volatile pesticides and UHPLC-MS/MS for polar and thermolabile compounds. The analytical method developed in this study can provide reliable technical means and data support for the scientific selection of high-quality producing areas, standardized cultivation management, and whole-process quality control of Platycodonis Radix. Furthermore, it offers a technical basis for quality and safety supervision of Platycodonis Radix and related medicinal and edible products, thereby contributing to the advancement of the traditional Chinese medicine industry toward standardization, regulation, and sustainable development.

## 2. Materials and Methods

### 2.1. Chemicals and Reagents

Acetonitrile and methanol (HPLC grade) were acquired from MREDA Inc. (Beijing, China). Formic acid (HPLC grade) was obtained from Macklin Biochemical Technology Co., Ltd. (Shanghai, China). Glacial acetic acid (analytical grade) was sourced from Shanghai Yuanye Bio-Technology Co., Ltd. (Shanghai, China). Organic microporous membranes (0.22 μm) were acquired from ANPEL Laboratory Technologies Inc. (Shanghai, China).

The QuEChERS dSPE kits used in this study were specifically designed for pesticide residue analysis in traditional Chinese medicinal materials according to the Chinese Pharmacopoeia. The kits, purchased from ANPEL Laboratory Technologies (Shanghai, China), included the SBEQ-CA8115-BZ dSPE extraction kit (AOAC 2007.01), with each unit containing 6 g magnesium sulfate (MgSO_4_) and 1.5 g sodium acetate (NaAc), and SBEQ-CA8773-25 Pharmacopoeia-oriented dSPE dispersive SPE purification tubes, each containing 900 mg MgSO_4_, 300 mg PSA (primary secondary amine), 30 mg C18, 30 mg graphitized carbon black (GCB), and 300 mg silica (Si). A Platycodonis Radix blank matrix (P/N 63102) was purchased from Shanghai Standard Technology Service Co., Ltd. (Shanghai, China).

### 2.2. Sample Collection

Platycodonis Radix samples were collected from major cultivation bases in China. A total of 42 samples were obtained from 9 different origins, and the specific information is listed in Table S1. All collected medicinal materials were peeled, dried in a far-infrared drying oven at 50 °C, ground, sieved with a No. 3 sieve, and stored in a dry place for further use.

### 2.3. Sample Preparation

The extraction procedure was performed using the QuEChERS method. Specifically, 3 g of pulverized Platycodonis Radix was accurately weighed into a 50 mL polypropylene centrifuge tube. Thereafter, 15 mL of 1% (*v*/*v*) glacial acetic acid solution was added, and the mixture was vortex-mixed for 1 min and allowed to stand for 30 min. Subsequently, 15 mL of acetonitrile was introduced, followed by vortex mixing and mechanical shaking for 8 min at 300 strokes/min to ensure complete sample homogenization. A QuEChERS extraction salt packet (containing MgSO_4_ and NaAc) was then added and manually dispersed, and the tube was vigorously shaken for 6 min under the same oscillation conditions.

The mixture was subsequently left to cool in an ice bath for 10 min and then centrifuged at 4000× *g* for 5 min at 4 °C. A 9 mL aliquot of the supernatant was transferred into a dSPE cleanup tube. The mixture was vortexed for 1 min and shaken vigorously for 8 min at 300 strokes/min. After centrifugation under the same conditions (4000× *g*, 5 min, 4 °C), 5 mL of the purified supernatant was transferred to a 10 mL glass tube and concentrated to approximately 0.4 mL under a gentle stream of nitrogen in a 40 °C water bath. The residue was reconstituted to 1 mL with acetonitrile and filtered through a 0.22 μm nylon syringe filter into an autosampler vial prior to chromatographic analysis.

### 2.4. Preparation of Stock and Working Solutions

A 1 mL aliquot of the mixed standard solution containing 69 pesticides (concentration range: 4–20 μg/mL) was accurately pipetted into a 20 mL volumetric flask. The solution was diluted to the mark with acetonitrile and thoroughly mixed. The resulting working standard solution was stored at −25 °C until further use.

### 2.5. GC-MS/MS Analyses

GC-amenable pesticides were determined using a Thermo Scientific TSQ 8000 triple quadrupole mass spectrometer (Thermo Fisher Scientific, Waltham, MA, USA) coupled with a Trace 1310 gas chromatograph and equipped with a TriPlus RSH autosampler (Thermo Fisher Scientific, Waltham, MA, USA). Chromatographic separation was achieved using a TG-17Sil MS capillary column (30 m × 0.25 mm, 0.25 μm, Thermo Scientific, USA). The column temperature was initially held at 60 °C for 1 min and then increased to 170 °C at a rate of 30 °C/min, followed by a gradual rise to 230 °C at 2 °C/min. Finally, the temperature was elevated to 300 °C at 15 °C/min and maintained for 6 min. The total run time was 45.33 min. High-purity helium (He) was used as the carrier gas, and the injector temperature was set at 250 °C. The sample was introduced in splitless mode with an injection volume of 1 μL.

The triple quadrupole MS was operated in selected reaction monitoring (SRM) mode and electron ionization (EI) mode, with the transfer line and ion source temperatures set to 250 °C.

### 2.6. UHPLC-MS/MS Analyses

An Agilent 1290 series II liquid chromatograph coupled with a 6475 triple quadrupole mass spectrometer (Agilent Technologies, Santa Clara, CA, USA) was employed for the analysis. Chromatographic separations were performed on a reversed-phase Agilent Zorbax Eclipse Plus C18 column (3.0 × 150 mm, 1.8 μm; Santa Clara, CA, USA). Mobile phase A consisted of 0.1% (*v*/*v*) formic acid aqueous solution containing 5 mmol/L ammonium formate. Mobile phase B was HPLC-grade methanol. A linear binary mobile phase solvent gradient was used as follows: 80% A at 0 min, 50% A at 2 min, 0% A at 12 min, 0% A at 15 min, and 80% A at 15.1 min. The flow rate, column temperature, and injection volume were 0.3 mL/min, 40 °C, and 1 μL, respectively.

The mass spectrometer was operated using electrospray ionization (ESI) in positive- and negative-ion modes. The ionization parameters were as follows: capillary voltage: 4 kV; drying gas temperature: 250 °C; drying gas flow rate: 7 L/min; sheath gas temperature: 350 °C; sheath gas flow rate: 12 L/min; nebulizer pressure: 350 kPa.

### 2.7. Pesticide Residue Risk Assessment

Pesticide residue risk was assessed using the hazard index method [24]. The estimated daily exposure was calculated according to the following equation:

(1) $$\mathrm{EXP}\;=\;\mathrm{Ef}\;\times \;\mathrm{Ed}\;\times \;\mathrm{IR}\;\times \;\frac{C}{\mathrm{AT}\;\times \;\mathrm{BW}}$$

where EXP represents the estimated daily pesticide exposure (mg/kg/bw); Ef (exposure frequency) was set as 90 days per year based on the P95 percentile from the national food consumption survey [25]; Ed (exposure duration) refers to the lifetime exposure to herbal medicine, assumed to be 20 years [26]; IR is the daily intake of herbal material (kg/d)—for short-term exposure assessment, the maximum daily intake at the P95 percentile (0.01 kg/d) was applied, while for long-term assessment, the mean daily intake (0.0065 kg/d) was used, both according to the Platycodonis Radix monograph in the Chinese Pharmacopoeia 2025 edition [2]; C denotes the measured concentration of pesticide residue in the herbal material (mg/kg); AT (averaging time) was set as 25,550 days, corresponding to a 70-year lifetime; and BW (body weight) was set at 63 kg.

(2) $$HQ_{a}=\frac{{\mathrm{EXP}}_{a}\;\times \;\mathrm{SF}}{\mathrm{ARfD}}$$

where HQ_a_ is the acute hazard quotient, where the subscript “a” refers to acute exposure; EXP_a_ represents the estimated short-term exposure (mg/kg); SF denotes the safety factor with a standard value of 100; and ARfD (acute reference dose) indicates the maximum acceptable daily intake for acute exposure (mg/kg).

(3) $$HQ_{c}=\frac{{\mathrm{EXP}}_{c}\;\times \;\mathrm{SF}}{\mathrm{ADI}}$$

where HQ_c_ is the chronic hazard quotient, where the subscript “c” refers to chronic exposure; EXP_c_ denotes the estimated long-term daily intake (mg/kg); and ADI (acceptable daily intake) refers to the maximum acceptable daily intake under chronic exposure (mg/kg).

(4) $$\mathrm{HI}=\sum_{i=1}^{n}HQ$$

where HQ (hazard quotient) denotes the risk quotient for an individual pesticide residue, and HI (hazard index) represents the cumulative risk index of multiple pesticide residues, both of which are utilized for assessing the long-term intake risk of pesticide residues in herbal medicines.

## 3. Results and Discussion

### 3.1. Optimizing the GC-MS/MS Parameters

**Figure 1:**
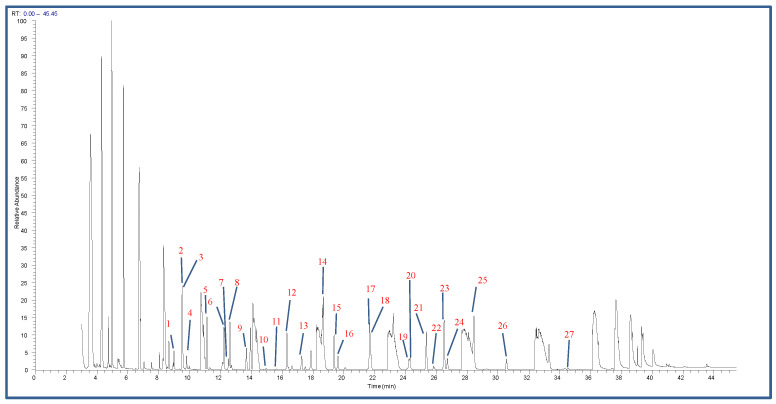
GC-MS/MS total ion chromatogram (TIC). The numbers 1–27 indicate the corresponding pesticide compounds listed in Table 1, and the blue leader lines indicate the corresponding chromatographic peaks.

During the development of the GC–MS/MS method, selected reaction monitoring mode was employed for analysis [27], in which one quantitative ion pair and two qualitative ion pairs were established for each of the 27 target pesticides; the optimized mass spectrometric parameters are listed in Table 1. The optimized total ion chromatogram (TIC) is shown in Figure 1. The results demonstrate that SRM mode enabled accurate identification of pesticides that were chromatographically co-eluted, thereby enhancing the specificity and reliability of the analytical method.

### 3.2. Optimizing the UHPLC-MS/MS Parameters

Three columns with different specifications were compared: the ZORBAX Eclipse Plus C18 column (3.0 × 150 mm, 1.8 μm), Hypersil GOLD C18 column (2.1 × 100 mm, 1.9 μm), and Hypersil GOLD column (2.1 × 150 mm, 3 μm). The results indicate that the ZORBAX Eclipse Plus C18 column provided the highest separation efficiency with a sharp peak shape and no apparent tailing or fronting. The mobile phase system was optimized with reference to the fifth method for pesticide residue determination specified in the Chinese Pharmacopoeia (2025 Edition), General Chapter 2341. The mobile phase in the reference method consisted of mobile phase A (0.1% aqueous formic acid containing 5 mmol/L ammonium formate) and mobile phase B (methanol–0.1% aqueous formic acid containing 5 mmol/L ammonium formate, 95:5, *v*/*v*). In this study, we optimized the mobile phase system by changing mobile phase B to HPLC-grade pure methanol while maintaining mobile phase A as 0.1% (*v*/*v*) aqueous formic acid containing 5 mmol/L ammonium formate, and adjusting the time program of linear binary gradient elution (consistent with the gradient program described in Section 2.6).

Under consistent experimental conditions, the optimized mobile phase system (mobile phase A: 0.1% aqueous formic acid containing 5 mmol/L ammonium formate; mobile phase B: pure methanol) was evaluated in comparison with the mobile phase system in the reference method (methanol–0.1% aqueous formic acid containing 5 mmol/L ammonium formate, 95:5, *v*/*v*). The findings demonstrate that the optimized mobile phase system (with pure methanol as mobile phase B) offered better separation performance and simpler operation, as it eliminated the need for preparing mixed solutions containing acid and salts, thereby streamlining the experimental procedure. From the perspective of column maintenance, pure methanol as mobile phase B may also reduce the risk of salt precipitation in the organic phase and simplify routine preparation.

**Figure 2:**
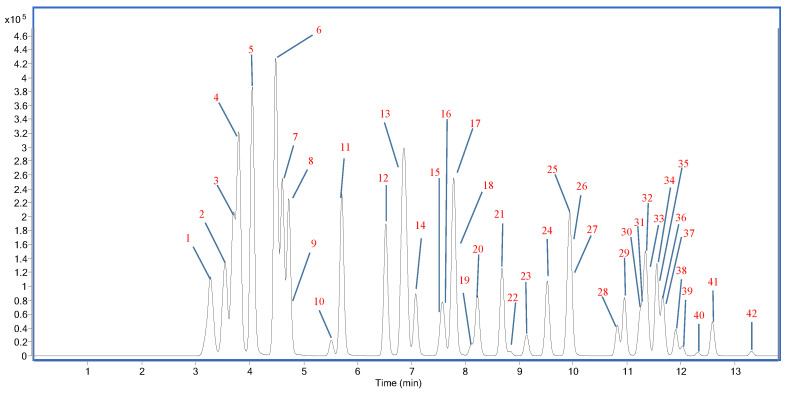
UHPLC-MS/MS total ion chromatogram (TIC). The numbers 1–42 indicate the corresponding pesticide compounds listed in Table 2, and the blue leader lines indicate the corresponding chromatographic peaks.

The mixed standard stock solution was diluted 20-fold with acetonitrile. Under the optimized chromatographic conditions described in Section 2.6, full-scan analyses were performed in both positive- and negative-ion modes to identify the precursor ions of the target compounds. Among them, fipronil and its three metabolites (Fipronil desulfinyl, Fipronil sulfone, Fipronil sulfide) consistently generated stable [M–H]^−^ ions in full-scan mode and were therefore analyzed in negative-ion mode. The remaining 38 pesticides were analyzed in positive-ion mode. Product ion scanning was then employed to screen characteristic product ions and explore appropriate collision energy ranges. Further optimization of mass spectrometric parameters—including fragmentor voltage, collision energy, and product ion selection—was conducted using multiple reaction monitoring mode. The final optimized MS parameters are summarized in Table 2. The optimized total ion chromatogram (TIC) is shown in Figure 2.

### 3.3. Method Validation

The method was validated following the SANTE 11312/2021 guideline document (SANTE/11312/2021, 2021) [28]. Although this guideline is primarily intended for pesticide analysis in food samples, it has been extensively adopted and proven applicable for similar multi-residue analysis in complex botanical matrices, including traditional Chinese medicines. Its validation protocols provide a comprehensive and accepted benchmark for ensuring method reliability. According to the SANTE guideline, a method must be tested for linearity, extraction recoveries, LODs and LOQs.

#### 3.3.1. Linearity and Limits of Detection and Quantification

Under the optimized chromatographic–mass spectrometric conditions, a series of matrix-matched standard solutions were prepared by diluting 50, 100, 150, 200, and 500 μL of the 20-fold diluted mixed standard working solution to 1 mL with the blank matrix solution. Matrix-matched calibration curves were constructed by plotting the peak area (y) against the mass concentration of each target pesticide (x, μg/L), and the corresponding regression equations were derived.

The limit of detection (LOD) was determined at a signal-to-noise ratio (S/N) of 3, and the limit of quantification (LOQ) at S/N ≥ 10. The results (Table 3 and Table 4) indicate that all 69 pesticides exhibited good linearity within their respective concentration ranges, with correlation coefficients (R^2^) greater than 0.99. The LODs ranged from 0.0058 to 2.7933 μg/kg, and the LOQs from 0.0196 to 9.3109 μg/kg, all of which were below the maximum residue limits specified in the current national standards. These findings demonstrate that the developed method possesses high sensitivity and is fully suitable for the trace-level determination of pesticide residues.

#### 3.3.2. Matrix Effect

In mass spectrometric analysis, the detection of target compounds in complex matrix samples is susceptible to matrix interference, often manifesting as signal enhancement or suppression—a phenomenon known as the matrix effect (ME) [29]. To evaluate its impact on detection outcomes, this study calculated the matrix effect by comparing the slope of the matrix-matched calibration curve, prepared using blank matrix extract, with that of the solvent-based (acetonitrile) calibration curve. The intensity of the matrix effect was classified as follows: an ME value between 20% and 50% indicated a moderate effect, below 20% a weak effect, and above 90% a strong effect [30].

**Figure 3:**
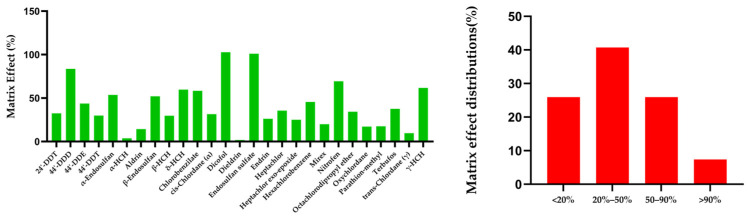
Matrix effects of the 27 pesticides detected via GC-MS/MS. The left panel shows the matrix effect (%) of each pesticide, and the right panel shows the distribution of pesticides across different matrix-effect ranges.

**Figure 4:**
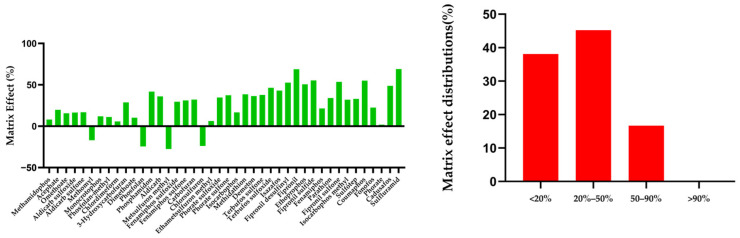
Matrix effects of the 42 pesticides detected via UHPLC-MS/MS. The left panel shows the matrix effect (%) of each pesticide, and the right panel shows the distribution of pesticides across different matrix-effect ranges.

The results demonstrate (Figure 3 and Figure 4) that among the 27 pesticides analyzed via GC-MS/MS, the matrix effect ranged from 1.83% to 102.76%, with 2 exhibiting a strong effect, 18 a moderate effect, and 7 a weak effect. For the 42 pesticides analyzed via UHPLC-MS/MS, the matrix effect ranged from −24.58% to 69.12%, with 17 showing a weak effect and 25 a moderate effect. To effectively mitigate interference from the matrix effect, all calibration standards in this experiment were prepared using a blank Platycodonis Radix matrix free of the target analytes.

#### 3.3.3. Extraction Recoveries

**Figure 5:**
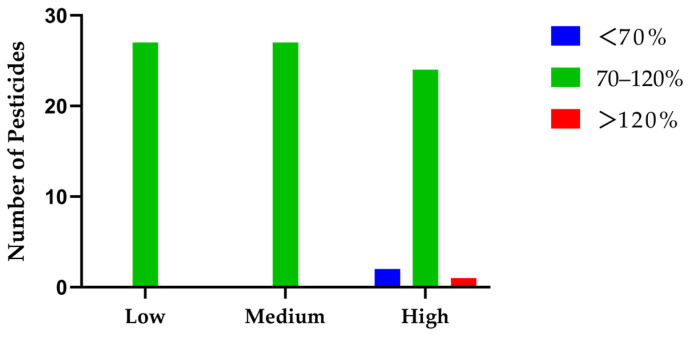
Recovery rates of the 27 pesticides (GC-MS/MS) at low, medium, and high spiked levels. Bars indicate the number of pesticides with recoveries of <70%, 70–120%, and >120%; absence of a bar indicates zero pesticides in that category.

**Figure 6:**
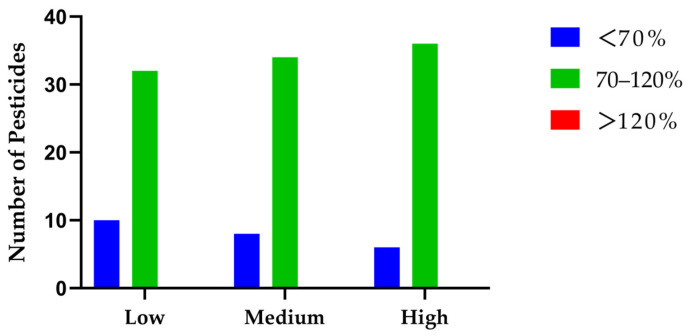
Recovery rates of the 42 pesticides (UHPLC-MS/MS) at low, medium, and high spiked levels. Bars indicate the number of pesticides with recoveries of <70%, 70–120%, and >120%; absence of a bar indicates zero pesticides in that category.

The accuracy and precision of the method were evaluated through recovery experiments. A 3 g aliquot of blank Platycodonis Radix matrix powder was fortified at three levels (low, medium, and high), corresponding to 0.5-, 1-, and 2-fold the reporting limit (RL). For each fortification level, six replicates were prepared according to the procedure outlined in Section 2.3. As presented in Figure 5 and Figure 6, the recovery rates for the majority of pesticides fell within the acceptable range of 70% to 120%. Specifically, the recoveries for all target compounds ranged from 60.05% to 120.44%, with associated relative standard deviations (RSDs) all below 10%. These data indicate that the method showed generally acceptable accuracy and good precision.

### 3.4. Application of the Analytical Method

**Figure 7:**
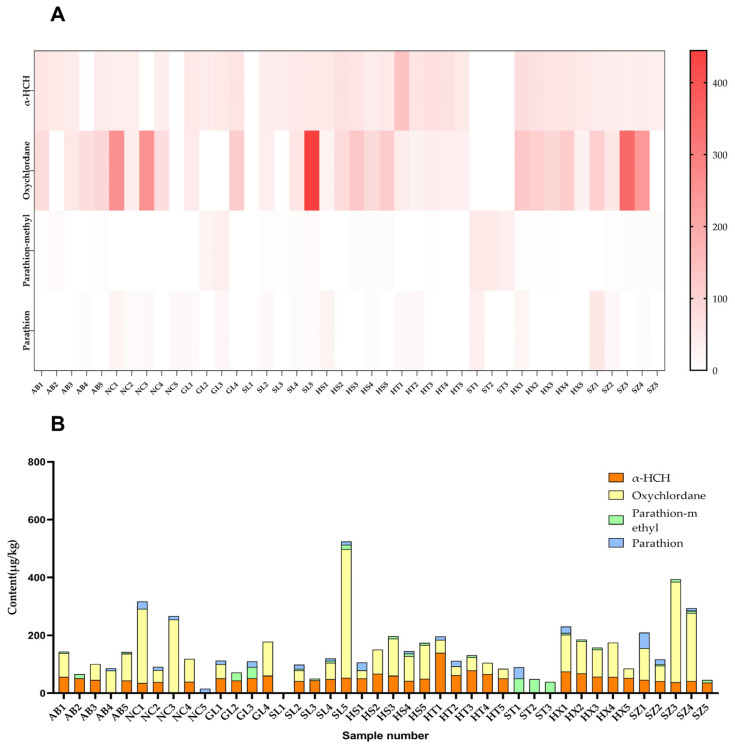
Residual concentrations of four pesticides in different samples. (**A**): Heatmap of residual concentrations. (**B**): Stacked bar chart of individual component concentrations.

The established method was applied to analyze 42 batches of Platycodonis Radix samples collected from different origins. The results are presented in Figure 7 and Table 5.

Among the 27 pesticide compounds monitored via GC-MS, 3 were detected: α-HCH was found in 35 batches at concentrations ranging from 0.035 to 0.14 mg/kg, 1 batch exceeded the ChP MRL (0.1 mg/kg), and 20 batches exceeded the GB 2763 MRL (0.05 mg/kg). No MRLs are specified by the EU or CAC; oxychlordane was detected in 32 batches within the range of 0.028–0.44 mg/kg, 12 batches exceeded the ChP MRL (0.1 mg/kg), and 32 batches exceeded the GB 2763 MRL (0.02 mg/kg). No MRLs are specified by the EU or CAC; parathion-methyl was identified in 24 batches at levels between 0.0048 and 0.051 mg/kg, 5 batches exceeded the MRLs set by ChP, GB 2763, and the EU (all at 0.02 mg/kg), while no batches exceeded the CAC MRL (3 mg/kg). Among the 42 pesticides analyzed via UHPLC-MS, only Parathion was detected, present in 19 batches at concentrations of 0.0076–0.054 mg/kg, 5 batches exceeded the MRLs of ChP and the EU (both 0.02 mg/kg), 7 batches exceeded the GB 2763 MRL (0.01 mg/kg), and no batches exceeded the CAC MRL (0.2 mg/kg).

These findings indicate that pesticide residues or pesticide-related environmental contamination remain present in some Platycodonis Radix samples and producing areas. Among the detected compounds, α-HCH and oxychlordane belong to the organochlorine pesticide class. China has historically been a major producer and user of organochlorine pesticides, which, while effectively improving crop yields, have also significantly contaminated soil and water systems [31,32]. In this study, α-HCH and oxychlordane were detected in the majority of samples, with particularly high concentrations observed in certain batches (Figure 7). This widespread occurrence suggests that their presence is likely derived from not only historical agricultural practices but also persistent residual contamination in the soil and water environments of major Platycodonis Radix-producing areas, rather than recent direct application.

Notably, the distribution patterns of these pesticides varied among different geographical origins. Oxychlordane exhibited notably high concentrations in samples from several origins, with some batches exceeding the regulatory MRL, indicating that the planting environments in these regions may have a higher legacy pollution burden from organochlorine pesticides. Similarly, α-HCH showed widespread but uneven distribution across samples, with a few batches reaching concentrations close to or above the limit, reflecting regional differences in historical pollution levels and environmental persistence. In contrast, methyl Parathion and Parathion were detected in fewer batches and at lower concentrations, but their presence in multiple origins suggests potential cross-contamination from adjacent agricultural fields or non-targeted pesticide use during cultivation.

Organochlorine pesticides are highly persistent and bioaccumulative, leading to long-term retention in human adipose tissue, liver, and other organs with limited metabolic clearance. Prolonged exposure may elevate the risks of hepatic injury and hematological disorders [33,34]. Methyl Parathion and Parathion are organophosphorus pesticides, characterized by high acute toxicity but relatively rapid degradation [35]. Their health hazards are primarily associated with acute neurotoxic effects, including symptoms such as sweating, tremors, and confusion upon high-dose exposure, which can progress to respiratory paralysis and even death in severe cases [36].

The uneven distribution of these pesticide residues across different producing areas highlights the need for origin-specific quality control strategies. Excessive or irrational pesticide application not only harms the environment and ecosystems but also poses direct threats to human health, especially considering the long-term medicinal and dietary use of Platycodonis Radix. Therefore, strengthening, environmental monitoring of historical pollution in major producing areas, promoting standardized cultivation practices that minimize cross-contamination, and establishing targeted quality testing protocols for high-risk origins are crucial steps to ensure the safety of Platycodonis Radix. Concurrently, enhancing the quality and safety testing services for agricultural products and establishing a comprehensive quality and safety control system throughout the production chain have become urgent priorities to reduce dietary exposure risks and ensure the sustainable development of the Platycodonis Radix industry.

### 3.5. Risk Assessment

The results indicate (Table 6 and Table 7) that the chronic/acute exposure risks for all pesticides detected were significantly below 1. Therefore, long-term exposure to these pesticides through the consumption of Platycodonis Radix poses no significant health risk to humans. Note that MRL exceedance and dietary risk assessment represent different evaluation perspectives. The former indicates potential non-compliance in quality control, whereas HI values below 1 suggest that the estimated dietary exposure risk under the assumed consumption scenarios remains low. This conclusion underscores the overall safety of the analyzed samples concerning pesticide residues under the current usage patterns. However, continuous monitoring and adherence to Good Agricultural Practices (GAPs) remain essential to ensure the sustained quality and safety of herbal materials. Future studies could expand the risk assessment to include combined exposure effects from multiple pesticide residues and different consumption scenarios.

## 4. Conclusions

This study developed an applicable analytical method for the determination of 69 pesticide residues in Platycodonis Radix using a QuEChERS-based extraction procedure coupled with gas chromatography–tandem mass spectrometry (GC–MS/MS) and liquid chromatography–tandem mass spectrometry (UHPLC–MS/MS). The method demonstrated good linearity over the concentration range of 4–500 ng/mL, with correlation coefficients (R^2^) exceeding 0.99 for all target pesticides. The mean recoveries at low, medium, and high spiked levels ranged from 60.05% to 120.44%, and the relative standard deviations (RSDs) were between 0.28% and 9.96%. The limits of detection (LODs) and quantification (LOQs) were 0.0058–2.7933 μg/kg and 0.0196–9.3109 μg/kg, respectively.

The proposed workflow exhibited satisfactory sensitivity, precision, and accuracy and was suitable for the qualitative and quantitative analysis of the target pesticide residues in Platycodonis Radix. In the analysis of 42 batches of samples, four pesticides were detected, and the dietary risk assessment showed that both the acute and chronic hazard index values were below 1, indicating that the estimated dietary exposure risks were low under the assumed consumption scenarios. Therefore, this validated workflow may provide a useful analytical approach for pesticide residue monitoring, quality control, and dietary risk assessment of Platycodonis Radix. However, several limitations should be acknowledged. First, the QuEChERS procedure was not systematically optimized in terms of extraction and cleanup conditions. Second, the present workflow was not directly compared with other sample preparation methods (e.g., SPE, GPC, and ASE) or other instrumental analytical methods (e.g., GC-IT-MS and GC-FPD). Further studies involving systematic method optimization and direct comparison with general/reference methods would be valuable for more comprehensive evaluations of the relative performance, robustness, and broader applicability of this workflow.

## Figures and Tables

**Table 1:** GC-MS/MS parameters for the detection of 27 pesticide compounds.

NO.	Pesticides	Retention Time (min)	Quantitative Ion Pair and Collision Energy (V)	Qualitative Ion Pair and Collision Energy (V)	
				1	2
1	Hexachlorobenzene	9.06	283.9 > 213.9, 32	142.0 > 107.0, 24	285.9 > 250.9, 16
2	α-HCH	9.58	181.0 > 145, 14	111.0 > 75.1, 12	183.0 > 147.0, 14
3	δ-HCH	9.58	181.0 > 145.0, 14	111.0 > 75.1, 12	183.0 > 147.0, 14
4	Terbufos	9.84	231.0 > 128.9, 24	97.0 > 79.0, 14	57.1 > 29.2, 10
5	β-HCH	11.18	181.0 > 145.0, 14	111.0 > 75.1, 12	183.0 > 147.0, 14
6	Heptachlor	12.26	100.1 > 65.1, 12	65.1 > 39.1, 10	271.9 > 236.9, 16
7	γ-HCH	12.68	181.0 > 145.0, 14	111.0 > 75.1, 12	183.0 > 147.0, 14
8	Octachlorodipropyl ether	12.76	79.0 > 49.0, 12	130.0 > 95.0, 22	131.9 > 97.0, 22
9	Aldrin	13.73	66.1 > 65.1, 8	262.9 > 192.9, 30	264.9 > 229.9, 16
10	Parathion-methyl	15.02	109.1 > 79.0, 8	125.0 > 79.1, 8	263.0 > 109.2, 12
11	Dicofol	15.7	139.1 > 111.1, 12	141.0 > 113.0, 12	75.1 > 74.1, 14
12	Oxychlordane	16.35	149.0 > 121.0, 6	115.0 > 87.0, 10	185.0 > 121.0, 10
13	Heptachlor exo-epoxide	17.3	81.1 > 53.1, 10	139.1 > 111.0, 12	236.9 > 142.9, 22
14	trans-Chlordane (γ)	18.68	136.1 > 108.1, 14	121.1 > 93.0, 8	236.9 > 142.8, 24
15	cis-Chlordane (α)	19.41	372.9 > 266.0, 24	236.9 > 118.9, 22	65.2 > 39.1, 10
16	α-Endosulfan	19.65	195.0 > 159.1, 12	170.0 > 99.1, 28	236.9 > 165.0, 26
17	4,4′-DDE	21.72	246.0 > 176.1, 28	176.1 > 150.1, 20	248.0 > 176.0, 32
18	Dieldrin	21.81	79.1 > 77.1, 12	77.1 > 51.1, 12	81.1 > 53.1, 12
19	Endrin	24.26	262.9 > 193.0, 30	139.1 > 111.1, 12	81.1 > 53.1, 10
20	Chlorobenzilate	24.34	139.0 > 111.0, 12	111.0 > 75.1, 12	251.1 > 139.0, 14
21	2,4′-DDT	25.37	235.1 > 165.1, 22	165.1 > 164.2, 18	237.1 > 165.1, 20
22	Nitrofen	25.98	283.0 > 253.1, 10	139.1 > 113.0, 18	63.1 > 62.1, 12
23	4,4′-DDD	26.55	235.0 > 165.1, 22	165.1 > 164.2, 16	237.0 > 165.1, 22
24	β-Endosulfan	26.77	195.0 > 159.0, 10	170.0 > 99.2, 28	236.9 > 165.1, 22
25	4,4′-DDT	28.47	235.0 > 165.1, 22	165.1 > 164.2, 18	237.0 > 165.1, 22
26	Endosulfan sulfate	30.61	271.9 > 236.9, 12	238.9 > 204.1, 10	236.9 > 140.9, 16
27	Mirex	34.57	271.9 > 236.9, 12	236.9 > 118.9, 24	273.8 > 238.7, 16

**Table 2:** UHPLC-MS/MS parameters for the detection of 42 pesticide compounds.

NO.	Pesticides	Retention Time (min)	Quantitative Ion Pair and Collision Energy (V)	Qualitative Ion Pair and Collision Energy (V)	
				1	2
1	Methamidophos	3.31	142.0 > 94.0, 80	142.0 > 125.0, 80	/
2	Acephate	3.58	184.0 > 125.0, 60	184.0 > 143.0, 80	/
3	Omethoate	3.74	214.1 > 125.0, 80	214.1 > 183.0, 80	/
4	Aldicarb-sulfoxide	3.84	207.1 > 89.0, 80	207.1 > 132.0, 80	/
5	Aldicarb sulfone	4.11	223.1 > 76.0, 110	223.1 > 86.0, 110	240.1 > 86.0, 110
6	Methomyl	4.54	163.0 > 88.0, 80	163.0 > 106.0, 80	/
7	Monocrotophos	4.66	224.1 > 127.0, 100	224.1 > 193.0, 100	/
8	Phosfolan-methyl	4.78	228.0 > 109.0, 90	228.0 > 168.0, 90	/
9	Chlordimeform	4.85	197.1 > 46.0, 120	197.1 > 89.0, 120	197.1 > 117.0, 30
10	3-Hydroxycarbofuran	5.59	238.1 > 163.0, 80	238.1 > 181.0, 80	238.1 > 220.1, 80
11	Dimethoate	5.82	230.0 > 171.0, 80	230.0 > 199.0, 80	/
12	Phosfolan	6.61	256.0 > 140.0, 100	256.0 > 168.0, 100	256.0 > 228.0, 100
13	Phosphamidon	6.91	300.1 > 127.0, 120	300.1 > 174.0, 120	/
14	Aldicarb	7.00	208.1 > 89.0, 65	208.1 > 116.0, 65	/
15	Metsulfuron-methyl	7.60	382.0 > 167.1, 90	328.0 > 199.0, 90	/
16	Fenamiphos sulfoxide	7.67	320.1 > 171.0, 120	320.1 > 233.0, 120	320.1 > 292.0, 120
17	Fenamiphos sulfone	7.87	336.1 > 188.0, 125	336.1 > 266.0, 125	/
18	Carbofuran	7.89	222.1 > 123.1, 100	222.1 > 165.1, 100	/
19	Chlorsulfuron	8.12	358.0 > 141.0, 120	358.0 > 167.0, 120	/
20	Ethametsulfuron-methyl	8.36	411.1 > 168.0, 120	411.1 > 196.0, 120	/
21	Phorate sulfoxide	8.81	277.0 > 97.0, 80	277.0 > 143.0, 80	277.0 > 171.0, 80
22	Phorate sulfone	8.97	293.0 > 115.0, 60	293.0 > 171.0, 60	293.0 > 247.0, 60
23	Isocarbophos	9.26	312.0 > 236.0, 100	312.0 > 270.0, 100	/
24	Methidathion	9.66	303.0 > 85.0, 80	303.0 > 145.0, 80	/
25	Demeton	10.01	259.1 > 61.0, 100	259.2 > 89.0, 100	/
26	Terbufos sulfone	10.02	321.0 > 97.0, 120	321.0 > 171.0, 120	/
27	Terbufos sulfoxide	10.06	305.1 > 97.0, 60	305.1 > 187.0, 60	/
28	Isazafos	10.93	314.0 > 120.0, 100	314.0 > 162.0, 100	/
29	Fipronil desulfinyl	11.07	386.9 > 282.0, 100	386.9 > 351.0, 100	/
30	Fipronil	11.33	434.9 > 250.0, 120	434.9 > 330.0, 120	/
31	Ethoprophos	11.34	243.1 > 97.0, 100	243.1 > 131.0, 100	243.1 > 173.0, 100
32	Fipronil sulfide	11.44	418.9 > 262.0, 110	418.9 > 383.0, 110	/
33	Fenamiphos	11.48	304.1 > 202.0, 140	304.1 > 217.0, 140	304.1 > 234.1, 140
34	Parathion	11.66	292.0 > 236.0, 120	292.0 > 264.0, 120	/
35	Fipronil sulfone	11.67	450.9 > 282.0, 135	450.9 > 415.0, 135	/
36	Isocarbophos-methyl	11.75	332.0 > 121.0, 90	332.0 > 231.0, 90	332.0 > 273.0, 90
37	Sulfotep	11.77	323.0 > 97.0, 120	323.0 > 115.0, 120	323.0 > 171.0, 120
38	Coumaphos	12.01	363.0 > 227.0, 120	363.0 > 307.0, 120	/
39	Fonofos	12.13	247.0 > 109.1, 80	247.0 > 137.1, 80	/
40	Phorate	12.43	261.0 > 47.0, 70	261.0 > 75.0, 70	/
41	Cadusafos	12.67	271.1 > 97.0, 80	271.1 > 131.0, 80	271.1 > 159.0, 80
42	Sulfluramid	13.40	526.0 > 169.0, 160	526.0 > 219.0, 160	/

**Table 3:** Method validation results for 27 pesticides in GC-MS/MS analysis.

Pesticides	Calibration Curve	Linear r2	Linear Range (ng/mL)	LOD (μg/kg)	LOQ (μg/kg)	Recovery (%)			Repeatability, % RSD			ME (%)
						Low	Medium	High	Low	Medium	High	
Hexachlorobenzene	y = 1767.5x − 629.4	0.9961	4–100	0.2989	0.9963	93.10	90.92	82.71	7.5693	7.8146	8.7716	45.57
α-HCH	y = 339.1x + 4.2	0.9980	20–500	1.3190	4.3966	95.46	104.56	97.28	3.0184	6.1493	3.4101	3.63
δ-HCH	y = 2054.8x − 13,239.0	0.9981	20–500	1.0523	3.5075	104.19	107.74	97.24	4.0447	4.6947	8.1929	59.73
Terbufos	y = 2921.3x − 4885.2	0.9980	4–100	0.3622	1.2074	93.19	102.87	99.13	1.9742	3.2914	3.4123	37.57
β-HCH	y = 2558.2x − 16,950.0	0.9974	20–500	2.7586	9.1954	98.18	102.29	97.24	3.9500	3.3365	3.2164	29.63
Heptachlor	y = 3865.1x − 13,266.0	0.9985	10–250	0.6402	2.1340	92.64	95.63	91.09	2.7013	4.8958	3.8789	35.57
γ-HCH	y = 194.7x + 215.0	0.9960	20–500	0.8247	2.7491	94.42	104.23	100.27	2.2264	3.9292	3.7558	61.70
Octachlorodipropyl ether	y = 2093.6x − 3573.6	0.9981	4–100	0.4845	1.6149	95.09	107.73	95.30	2.1736	3.5528	6.0079	34.29
Aldrin	y = 574.5x − 1612.5	0.9995	10–250	1.8204	6.0680	95.55	77.80	108.19	9.8979	2.5176	3.4616	14.27
Parathion-methyl	y = 1683.3x − 5126.0	0.9972	4–100	0.7160	2.3866	91.83	103.28	120.44	2.5976	5.8234	7.3344	17.51
Dicofol	y = 165.9x + 2526.2	0.9881	4–100	0.5658	1.8859	97.65	107.15	101.10	2.0197	7.2231	3.8249	102.76
Oxychlordane	y = 1173.6x − 13,151.0	0.9961	20–500	1.2508	4.1693	86.88	88.49	92.48	5.4079	3.9166	3.4170	17.17
Heptachlor exo-epoxide	y = 555.9x − 4199.5	0.9962	10–250	1.6547	5.5157	112.32	108.26	69.13	4.7242	7.2043	7.2055	25.02
trans-Chlordane (γ)	y = 766.4x − 6184.4	0.9974	20–500	2.1622	7.2072	100.13	107.90	100.87	1.9672	3.4806	4.7888	9.70
cis-Chlordane (α)	y = 787.0x − 6531.1	0.9986	20–500	2.7015	9.0050	95.98	94.79	91.98	2.0306	4.6125	5.0554	31.59
α-Endosulfan	y = 447.3x − 2541.9	0.9980	10–250	2.7933	9.3110	96.34	95.60	109.02	9.9602	8.2000	4.9355	53.63
4,4′-DDE	y = 4297.6x − 6539.5	0.9979	20–500	0.5299	1.7665	97.32	88.38	90.94	2.2477	5.3730	3.9884	43.73
Dieldrin	y = 329.3x − 2391.0	0.9937	10–250	2.5084	8.3612	70.46	106.90	79.05	7.2211	9.1227	7.2179	1.83
Endrin	y = 398.7x − 3254.2	0.9994	10–250	2.7397	9.1324	98.86	100.01	95.18	5.0103	3.5718	3.0654	26.22
Chlorobenzilate	y = 5394.8x − 4961.1	0.9988	10–250	0.5201	1.7337	97.76	109.90	106.65	2.9734	5.5055	4.4307	58.33
2,4′-DDT	y = 5277.5x − 32,902.0	0.9965	20–500	0.4152	1.3841	98.39	87.24	89.08	3.2908	9.0795	5.3690	32.38
Nitrofen	y = 1179.9x − 10,358.0	0.9968	10–250	1.4218	4.7393	95.57	112.03	106.49	2.4172	5.0886	6.2379	69.27
4,4′-DDD	y = 9006.3x − 17,829.0	0.9960	20–500	0.3563	1.1878	97.68	105.33	99.32	1.7184	5.5988	3.6595	83.55
β-Endosulfan	y = 494.5x − 4324.4	0.9956	10–250	1.3793	4.5977	106.96	108.43	96.92	5.0383	4.9671	3.9683	52.06
4,4′-DDT	y = 3183.7x − 23,798.0	0.9968	20–500	0.1845	0.6151	102.22	96.15	90.20	8.3384	6.6958	7.4234	29.95
Endosulfan sulfate	y = 1614.3x − 4951.0	0.9982	10–250	0.6529	2.1763	101.52	109.96	96.49	3.1242	5.1162	6.5594	101.10
Mirex	y = 3867.2x − 4208.2	0.9980	2–50	0.4444	1.4815	95.85	70.06	69.35	3.0953	4.9632	6.8593	19.98

**Table 4:** Method validation results for 42 pesticides in UHPLC-MS/MS analysis.

Pesticides	Calibration Curve	Linear r2	Linear Range (ng/mL)	LOD (μg/kg)	LOQ (μg/kg)	Recovery (%)			Repeatability, %RSD			ME (%)
						Low	Medium	High	Low	Medium	High	
Methamidophos	y = 2099.2x − 2925.0	0.9988	10–250	0.1586	0.5288	62.03	65.85	60.87	1.1610	0.3931	0.9561	8.10
Acephate	y = 2678.6x − 6253.1	0.9984	10–250	0.6736	2.2452	63.17	66.00	62.50	0.8052	1.1499	1.5546	19.77
Omethoate	y = 2270.0x − 4264.7	0.9988	10–250	0.0059	0.0196	83.47	89.72	83.12	2.7526	0.7831	0.7813	15.66
Aldicarb sulfoxide	y = 1266.7x − 4001.8	0.9991	20–500	0.0257	0.0856	79.32	85.31	96.27	4.7910	0.4489	3.5245	16.51
Aldicarb sulfone	y = 1003.9x − 1368.0	0.9996	20–500	0.0365	0.1218	63.80	69.77	64.67	1.2925	1.1238	1.4495	16.99
Methomyl	y = 732.7x − 4071.9	0.9986	20–500	0.0479	0.1595	68.05	92.74	60.05	1.7282	2.8155	1.5290	−16.92
Monocrotophos	y = 2481.6x − 3567.2	0.9988	6–150	0.0100	0.0332	89.76	64.25	78.31	3.7893	0.6128	0.7295	11.98
Phosfolan-methyl	y = 2758.6x − 3819.9	0.9975	6–150	0.0438	0.1458	63.95	66.07	77.52	1.7462	1.0428	0.3421	11.18
Chlordimeform	y = 881.8x − 1007.6	0.9973	4–100	0.0142	0.0474	63.77	64.97	99.78	0.6325	1.0605	0.9600	5.84
3-Hydroxycarbofuran	y = 431.7x + 491.9	0.9971	4–100	0.0716	0.2388	89.15	87.00	78.52	2.0910	1.5404	0.6614	28.85
Dimethoate	y = 2465.2x − 6109.8	0.9985	10–250	0.0427	0.1423	63.53	64.53	61.07	0.9888	1.0495	0.6356	10.32
Phosfolan	y = 2408.9x − 3606.3	0.9990	6–150	0.0227	0.0757	105.01	90.43	99.67	1.5330	0.4929	0.9124	−24.40
Phosphamidon	y = 2211.5x − 5955.7	0.9954	10–250	0.0167	0.0555	86.32	87.77	85.35	1.0878	0.9524	0.6741	41.87
Aldicarb	y = 781.3x − 3695.5	0.9982	2–50	0.0263	0.0876	70.55	76.98	72.33	1.1152	1.2471	0.8045	36.01
Metsulfuron methyl	y = 1815.2x − 1943.4	0.9990	4–100	0.1449	0.4832	83.78	85.87	83.90	1.2908	2.1330	1.1605	−27.56
Fenamiphos sulfoxide	y = 1675.5x − 1549.3	0.9968	4–100	0.0268	0.0892	77.07	80.20	75.58	1.5390	1.0462	1.0398	29.52
Fenamiphos sulfone	y = 2150.9x − 2049.8	0.9972	4–100	0.0178	0.0592	82.05	87.12	82.28	1.4436	0.6429	1.2894	31.15
Carbofuran	y = 4479.1x − 4884.8	0.9976	4–100	0.0095	0.0318	76.43	81.32	77.13	0.4872	0.6480	0.6745	32.19
Chlorsulfuron	y = 487.2x − 1303.4	0.9984	10–250	0.0544	0.1814	73.22	72.75	71.00	3.6906	1.7832	0.5270	−23.76
Ethametsulfuron methyl	y = 4333.9x − 16,102.7	0.9987	10–250	0.0121	0.0402	66.37	72.37	70.77	1.1502	0.5504	0.7131	6.37
Phorate sulfoxide	y = 3234.8x − 967.8	0.9982	2–50	0.0159	0.0530	78.73	83.12	80.07	0.9628	0.5561	0.3225	34.68
Phorate sulfone	y = 337.6x − 100.1	0.9962	2–50	0.1155	0.3851	81.60	84.27	79.35	8.3120	5.1068	4.0223	37.45
Isocarbophos	y = 192.0x − 493.8	0.9995	10–250	0.1562	0.5208	71.52	81.08	75.50	1.2171	1.2392	0.8122	16.74
Methidathion	y = 1792.6x − 3626.7	0.9986	10–250	0.0436	0.1454	64.75	85.73	81.42	1.5682	0.8270	1.0259	38.50
Demeton	y = 506.9x − 529.4	0.9979	4–100	0.0153	0.0509	74.10	74.87	71.98	1.7155	2.4841	0.7106	36.44
Terbufos sulfone	y = 1482.6x − 1323.3	0.9977	4–100	0.0185	0.0616	77.85	78.78	73.22	1.4042	0.3801	0.7649	37.84
Terbufos sulfoxide	y = 5450.0x − 5880.7	0.9967	4–100	0.0090	0.0298	80.55	82.57	78.30	0.4695	0.7724	0.7748	46.46
Isazafos	y = 2789.1x − 1511.6	0.9976	2–50	0.0191	0.0636	88.30	93.90	86.05	2.1666	0.9087	0.8009	43.06
Fipronil desulfinyl	y = 1862.9x − 1363.4	0.9981	4–100	0.0314	0.1046	103.38	105.63	103.18	1.4560	0.6844	0.3749	52.69
Fipronil	y = 181.4x − 158.1	0.9985	4–100	0.0220	0.0734	107.13	106.12	110.25	2.9409	3.0793	1.6643	68.98
Ethoprophos	y = 1566.3x − 1709.5	0.9987	4–100	0.0298	0.0993	81.43	89.97	84.45	1.7671	0.5520	1.7383	50.63
Fipronil sulfide	y = 1915.5x − 1565.5	0.9974	4–100	0.0115	0.0383	102.65	104.38	102.75	1.4668	1.0658	0.9669	55.35
Fenamiphos	y = 3612.6x − 3260.2	0.9995	4–100	0.0184	0.0613	82.77	87.80	83.82	1.8398	1.5732	0.9587	21.47
Parathion	y = 48.5x + 36.1	0.9997	4–100	0.0187	0.0622	97.70	107.52	102.92	9.6365	4.0675	4.8284	34.06
Fipronil sulfone	y = 2209.6x − 2320.4	0.9958	4–100	0.0186	0.0621	99.70	102.70	100.30	1.2876	1.2378	0.4965	53.51
Isocarbophos-methyl	y = 744.6x − 456.6	0.9993	4–100	0.0476	0.1587	89.10	91.72	84.63	3.2139	4.9962	3.5828	32.12
Sulfotep	y = 1667.7x − 1760.0	0.9986	4–100	0.0273	0.0909	81.80	89.45	82.80	1.3325	1.4451	0.7638	33.12
Coumaphos	y = 1537.5x − 3534.7	0.9981	10–250	0.0580	0.1932	73.52	86.07	82.57	1.9013	0.6997	1.1532	55.09
Fonofos	y = 323.5x − 304.5	0.9990	4–100	0.1046	0.3488	77.25	81.02	76.37	4.8950	4.8535	2.2386	22.54
Phorate	y = 276.7x − 64.6	0.9995	2–50	0.0286	0.0953	92.37	107.65	94.20	3.0543	2.3462	1.9860	−0.37
Cadusafos	y = 2726.6x − 2506.4	0.9997	4–100	0.0158	0.0527	73.63	93.43	87.22	1.1254	0.2845	0.4436	48.75
Sulfluramid	y = 87.5x + 70.2	0.9959	4–100	0.0212	0.0707	62.03	65.85	60.87	1.1610	0.3931	0.9561	69.12

**Table 5:** Determination results of pesticide residues in 42 batches of Platycodonis Radix.

Instrument	Pesticides	Detected (n) ^(a)^	Concentration (mg/kg)	ChP MRL (mg/kg) ^(b)^	GB 2763 MRL (mg/kg) ^(c)^	EU MRL (mg/kg) ^(d)^	CAC MRL (mg/kg) ^(e)^	Exceed (ChP) ^(f)^	Exceed (GB 2763) ^(g)^	Exceed (EU) ^(h)^	Exceed (CAC) ^(i)^
GC-MS/MS	α-HCH	35	0.035–0.14	0.1	0.05	-	-	1	20	-	-
GC-MS/MS	Oxychlordane	32	0.028–0.44	0.1	0.02	-	-	12	32	-	-
GC-MS/MS	Parathion-methyl	24	0.0048–0.051	0.02	0.02	0.02	3	5	5	5	0
UHPLC-MS/MS	Parathion	19	0.0076–0.054	0.02	0.01	0.02	0.2	5	7	5	0

^(a)^ Number of samples in which the pesticide was detected among the 42 batches of Platycodonis Radix samples. ^(b)^ Maximum residue limit specified in the Chinese Pharmacopoeia. ^(c)^ Maximum residue limit specified in GB 2763 National Food Safety Standard—Maximum Residue Limits for Pesticides in Food. ^(d)^ Maximum residue limit specified by the European Union (EU). ^(e)^ Maximum residue limit specified by the Codex Alimentarius Commission (CAC). ^(f)^ Number of samples exceeding the MRL set by the Chinese Pharmacopoeia. ^(g)^ Number of samples exceeding the MRL set by GB 2763. ^(h)^ Number of samples exceeding the EU MRL. ^(i)^ Number of samples exceeding the CAC MRL.

**Table 6:** Acute pesticide residue hazard index.

Pesticides	AB	NC	GL	SL	HS	HT	ST	HX	SZ
α-HCH	0.0045	0.0025	0.0058	0.0042	0.0061	0.0089	0.0000	0.0069	0.0046
Oxychlordane	0.0137	0.0282	0.0074	0.0240	0.0198	0.0087	0.0000	0.0216	0.0334
Parathion-methyl	0.0051	0.0011	0.0168	0.0068	0.0050	0.0083	0.0473	0.0035	0.0062
Parathion	0.0006	0.0048	0.0030	0.0025	0.0026	0.0023	0.0029	0.0016	0.0059
HI_a_ ^(a)^	0.0238	0.0367	0.0331	0.0376	0.0334	0.0282	0.0502	0.0337	0.0501

^(a)^ HI_a_, the acute hazard index, is the sum of the hazard quotients (HQs) of all detected pesticides per producing region.

**Table 7:** Chronic pesticide residue hazard index.

Pesticides	AB	NC	GL	SL	HS	HT	ST	HX	SZ
α-HCH	0.0053	0.0030	0.0069	0.0050	0.0072	0.0105	0.0000	0.0082	0.0054
Oxychlordane	0.0890	0.2824	0.0742	0.2405	0.1978	0.0872	0.0000	0.2163	0.3339
Parathion-methyl	0.0182	0.0038	0.0601	0.0244	0.0178	0.0297	0.1690	0.0123	0.0221
Parathion	0.0014	0.0118	0.0073	0.0061	0.0063	0.0055	0.0072	0.0040	0.0144
HI_c_ ^(a)^	0.1139	0.3011	0.1485	0.2760	0.2291	0.1329	0.1762	0.2408	0.3758

^(a)^ HI_c_, the chronic hazard index, is the sum of the chronic hazard quotients (HQs) of all detected pesticides per producing region.

## Data Availability

The original contributions presented in the study are included in the article/Supplementary Materials; further inquiries can be directed to the corresponding authors.

## Supplementary Materials

The following supporting information can be downloaded at https://www.mdpi.com/article/10.3390/foods15111835/s1: Table S1: Platycodonis Radix sample information.
