# Supplementary material for: Determination of 69 Pesticide Residues in 42 Batches of Platycodonis Radix and Dietary Risk Assessment Using Combined QuEChERS with GC-MS/MS and UHPLC-MS/MS
Source: Foods. 2026 May 22;15(11):1835. doi: 10.3390/foods15111835 (PMC13257400; doi:10.3390/foods15111835)
Supplement: Supplementary file 1 [file foods-15-01835-s001.zip › foods-4274312-supplementary.pdf]

Table S1 Platycodonis Radix sample information.

Sample source	Sample Batch	Platycodonis Radix morphology
Xixia County, Henan Province	5	root
Songxian County, Henan Province	5	root
Chifeng City, Inner Mongolia Autonomous Region	5	root
Bozhou City, Anhui Province	5	root
Zibo City, Shandong Province	5	root
Shangluo City, Shaanxi Province	5	root
Longnan City, Gansu Province	4	root
Taiyuan City, Shanxi Province	3	root
Tongbai County, Henan Province	5	root
